# Oral Bacteriotherapy in Patients With COVID-19: A Retrospective Cohort Study

**DOI:** 10.3389/fnut.2020.613928

**Published:** 2021-01-11

**Authors:** Giancarlo Ceccarelli, Cristian Borrazzo, Claudia Pinacchio, Letizia Santinelli, Giuseppe Pietro Innocenti, Eugenio Nelson Cavallari, Luigi Celani, Massimiliano Marazzato, Francesco Alessandri, Franco Ruberto, Francesco Pugliese, Mario Venditti, Claudio M. Mastroianni, Gabriella d'Ettorre

**Affiliations:** ^1^Department of Public Health and Infectious Diseases, Sapienza University of Rome, Rome, Italy; ^2^Azienda Ospedaliero-Universitaria Policlinico Umberto I, Rome, Italy; ^3^Medical Statistics Unit, Department of Public Health and Infectious Diseases, Sapienza University of Rome, Rome, Italy; ^4^Department of Anaesthesia and Intensive Care, Sapienza University of Rome, Rome, Italy

**Keywords:** COVID-19, microbiota, gut, oral bacteriotherapy, pneumonia

## Abstract

**Background:** Mounting evidence suggests SARS-CoV-2 may impact on host microbiota and gut inflammation, infecting intestinal epithelial cells. This possible link and its implications can be investigated by observing the effects of modulation of the microbial flora in patients with COVID-19. The aim of this study was to compare the rate of mortality, the need of ICU hospitalization and the length of hospitalization in patients with severe COVID-19 pneumonia who received the best available therapy (BAT) vs. patients treated with BAT and supplemented with oral bacteriotherapy.

**Methods:** This retrospective, observational cohort study included 200 adults with severe COVID-19 pneumonia. All patients received therapeutic regimens including low molecular weight heparin plus one or more between hydroxychloroquine, azithromycin, antivirals, and Tocilizumab. Oral bacteriotherapy was used as complementary treatment.

**Results:** Out of the 200 patients, 112 received BAT without oral bacteriotherapy, and 88 BAT with oral bacteriotherapy. Crude mortality was 22%. Eleven percent died in the group of patients treated with BAT plus oral bacteriotherapy vs. 30% subjects in the group of patients managed only with BAT (*p* < 0.001). By multivariate analysis, the age >65 years, CRP >41.8 mg/L, Platelets <150.000 mmc, and cardiovascular events were associated with the increased risk of mortality. Oral bacteriotherapy was an independent variable associated with a reduced risk for death. Despite large prospective trials are needed, this study highlights a possible role for oral bacteriotherapy in the management of patients hospitalized for COVID-19 pneumonia.

## Introduction

Pneumonia and gastrointestinal symptoms as predominant clinical manifestations of COronaVIrus Disease-19 (COVID-19) are not accidental (1). Both lung and gut express the ACE2 receptor, through which the Severe Acute Respiratory Syndrome Coronavirus 2 (SARS-CoV-2) can enter cells. In the gut, where ACE2 receptors are abundantly present, coronavirus may multiply quickly, and a recent study found that the infection generally lasted longer in COVID-19 patients who had previously experienced gastrointestinal problems (2).

Older adults or subjects with diabetes mellitus (DM), hypertension, cerebrovascular disease, and chronic obstructive pulmonary disease (COPD) have a higher risk of death for severe COVID-19 infection (3, 4). The co-morbidities mentioned above present a common denominator: gut dysbiosis. Alterations of the gut microbiota composition as well as the loss of key intestinal bacterial species might be a facilitating factor for a dysregulated immune response against the SARS-CoV-2 (5). Therefore, the supplementation with beneficial microbes of the gastrointestinal tract of SARS-CoV-2 infected individuals seems not illogical.

We previously reported the results of a “real life” study analyzing the effects of an oral bacteriotherapy as support of standard of care treatment in patients hospitalized for SARS-CoV2 pneumonia and associated gastrointestinal symptoms (6). In particular, we observed that combined treatment may induce, within 72 h, remission of diarrhea, and other symptoms compared to less than half of the patients who received best available therapy (BAT) alone. Besides, the estimated risk of developing respiratory failure was eight-fold lower in patients also receiving oral bacteriotherapy (6).

Here we report the results of a more extensive “real life” study on the complementary use of a specific oral bacteriotherapy (Sivomixx® a multi-strain product containing five strains of lactobacilli, two strains of bifidobacteria, and one strain of *Streptococcus thermophilus*) in a cohort of individuals infected by SARS-CoV2 and receiving BAT.

## Materials and Methods

This is a retrospective observational “real-life” cohort study comparing the rate of crude mortality, the need of intensive care unit (ICU) hospitalization and the length of hospitalization in patients with severe COVID-19 pneumonia who received the best available therapy (BAT) vs. patients treated with BAT and supplemented with oral bacteriotherapy. Patients admitted in wards of the Department of Infectious Diseases of Policlinico Umberto I Hospital, Sapienza University of Rome, Italy, from 6th March until 26th April 2020 were evaluated. The Ethics Committee of Policlinico Umberto I approved the study (approval number/ID Prot. 109/20209).

### Patients

Diagnosis of SARS-CoV-2 was defined as one positive oropharyngeal and nasopharyngeal swab for COVID-19. It was performed in duplicate for SARS-CoV-2 E and S gene by a reverse transcriptase polymerase chain reaction (RT-PCR) (7). Studied population included subjects older than 18 years.

All the hospitalized patients received therapeutic regimens including hydroxychloroquine (200 mg twice a day for 7 days), azithromycin (500 mg once a day for 7 days), lopinavir–ritonavir (400/100 mg twice a day) or darunavir–cobicistat (800/150 mg once a day) for 14 days, and low molecular weight heparin for prophylaxis of deep vein thrombosis as recommended at the time by the Italian Society of Infectious Diseases (8). Tocilizumab (8 mg/kg up to a maximum of 800 mg per dose with an interval of 12 h for two times) was administered in case of high levels of serum IL-6 or of significant worsening of the respiratory picture in case of unavailability of IL-6 dosage. Patients admitted to the ward in case of intestinal symptoms received in addition to BAT, also supplementation with oral bacteriotherapy. The commercial formulation used was composed of *Streptococcus thermophilus* DSM 32245, *Bifidobacterium lactis* DSM 32246, *Bifidobacterium lactis* DSM 32247, *Lactobacillus acidophilus* DSM 32241, *Lactobacillus helveticus* DSM 32242, *Lactobacillus paracasei* DSM 32243, *Lactobacillus plantarum* DSM 32244, and *Lactobacillus brevis* DSM 27961 (Sivomixx®). The formulation was administered in three equal doses per day, for a total of 2,400 billion bacteria per day.

The data source for patient information analysis was derived from electronic medical records in the Hospital Electronic Information System. The variables considered for the study included: (1) age, gender, admission and discharge date from the hospital, length of stay (LOS); (2) cardiovascular (CV) disease, chronic lung disease, chronic kidney disease (CKD), hypertension, asthma, chronic obstructive pulmonary disease (COPD), diabetes mellitus, immunodeficiency, cancer (defined as active or past/resolved).

We used the Charlson score (9) to predict the 1-year mortality for a patient with a range of comorbid conditions. Confusion—Blood urea—Respiratory rate—Blood pressure score (CURB) (10), Confusion—Blood urea—Respiratory rate—Blood pressure, Age 65 (CURB-65) (11), Confusion—Blood urea—Respiratory rate—Blood pressure, Age 65, Lactate dehydrogenase, Platelet, and Albumin (expanded-CURB-65) (12), Pneumonia Severity Index (PSI) score (13) were used to define the severity of pneumonia. To predict the progression of COVID-19 we considered Comorbidity—Age—Lymphocyte—Lactate Dehydrogenase (CALL) (14).

### Statistical Analysis

The statistical analyses were conducted with Statistical package for social science (SPSS) software, version 22 (IBM SPSS, Chicago, III). The continuous data were presented as medians (IQR, 25°-75°) and the presence of statistically significant differences between groups were assessed by the nonparametric Mann–Whitney *U*-test. The dichotomous variables were described as simple frequencies and percentages (%) and then compared by the χ^2^ test for the two groups. A multivariate analysis of gradual regression was run with different factors potentially confounding, with Age > 65 years, Lymphocytes <1,000 in 1 μL of blood, platelets <150–103/mm^3^, albumin <32 g/dL, CV events, BAT therapy and oral antibacterial therapy as dependent variable. We have done a standard survival analysis, tracing participants from entry into the clinic to discharge or death. The event-free survival in follow-up was depicted graphically by Kaplan–Meier's survivor curve, using multivariable Cox regression analysis, including the confusion factors with fixed baseline covariates. The effect of treatment was shown using an unadjusted odds ratio (OR) adjusted with 95% CI. Principal sources of confusion were identified as age, C-reactive protein (CPR), Charlson's comorbidity index, CURB, CURB-65, PSI, Call, number of lymphocytes, and number of platelets the most likely causes of both treatment assignment and risk of the outcome. A two-sided *p*-value test of < 0.05 was considered statistically significant.

## Results

The data utilized are from the patients admitted in wards of the Department of Infectious Disease from March 6 until April 26, with the diagnosis of SARS-CoV-2 infection. Two hundred patients were included; the demographic and clinical characteristics of whole population enrolled were reported in Table 1.

**Table 1:** Demographic and clinical characteristics of whole population enrolled.

**Parameters**	**Mean (±SD)**	**Median (IQR 25–75%)**	**Number (%)**
Gender, Male/Female	–	–	113 (57)/87 (43)
Age	63 (±15)	63 (54–75)	–
	Male 62 (±15)		
	Female 64 (±16)		
White blood cells	6,261.5 (±2,766.4)	5,660 (4,420–7,205)	–
Neutrophils (mmc)	4,606.7 (±2,641.8)	4,050 (2,795–5,664)	–
Neutrophils (%)	70.8 (±13.1)	72.3 (63.3–81)	–
Lymphocytes (mmc)	1,083.4 (±649.2)	920.0 (670.0–1,330)	–
Lymphocytes (%)	19.2 (±10.6)	16.8 (11.5–29)	–
Monocytes (mmc)	362.2 (±160.6)	320.0 (250–470)	–
C-reactive protein (mg/L)	98,855.8 (±121,164.2)	53,165.0 (16,937.5–140,025)	–
D-dimer (mg/dl)	1,321.6 (±1,200.0)	878.0 (495.5–1,547)	–
Albumin (mg/dl)	36.9 (±5.7)	37.5 (33–42)	–
LDH (U/L)	333.0 (±168.5)	288.0 (226.5–391)	–
Platelets (mmc)	225,087 (±91,489)	208,000 (161,250–262,500)	–
Lenght of hospitalization	20 (±13.8)	15 (10–27)	–
CHARLSON index	2.6 (±2.2)	2.0 (1–4)	–
CURB-65	1.1 (±0.8)	1.0 (0–2)	–
EXP CURB 65	2.2 (±1.4)	2.0 (0–3)	–
PSI	73.4 (±29.6)	70.0 (51–89)	–
CALL	8.7 (±2.6)	9.0 (7–11)	–
Death	–	–	44 (22)

We are unable to report the duration of the symptoms before admission to the hospital. However, we are confident that the hospitalization took place within a few days after the onset of the respiratory symptoms since an efficient, free of charge, Health Service is present in the Lazio Region, Italy. The median length of hospitalization was 15 days [IQR, (10–27)]. Out of the 200 patients, 112 received BAT without oral bacteriotherapy while for 88 subjects, BAT was coupled with oral bacteriotherapy. The characteristics of the two groups are shown in Table 2. The oral bacteriotherapy was started after a median of 1 day (min 0, max 2) from the admission to the hospital.

**Table 2:** Population stratified in two groups according to the therapy: BAT or BAT plus oral bacteriotherapy.

**Parameters**	**BAT (*****n*** **=** **112)**			**BAT** **+** **oral bacteriotherapy (*****n*** **=** **88)**			***p*-value**
	**Mean (±SD)**	**Median (IQR 25–75%)**	**Number (%)**	**Mean (±SD)**	**Median (IQR 25–75%)**	**Number (%)**	
Gender, Male/Female	**–**	–	64 (57)/48 (43)	–	–	49 (56)/39 (44)	0.978
Age	64 (±16)	63 (55–75)	–	62 (±15)	63 (52–72)	–	0.289
Age > 65 years	–	–	53 (47)	–	–	38 (43)	0.448
Lymphocytes <1,000 (mmc)	–	–	60 (54)	–	–	49 (56)	0.898
PLT <150,000 (mmc)	–	–	16 (14)	–	–	22 (25)	0.718
Albumin <32 (mg/dl)	–	–	20 (18)	–	–	7 (8)	**0.024**
CRP >41.8 (mg/L)	–	–	64 (57)	–	–	40 (45)	0.055
White blood cells (mmc)	6,306 (±2,624)	5,740 (4,570–7,450)	–	6,206 (±2,944)	5,590 (4,327–7,155)	–	0.803
Neutrophils (mmc)	4,716 (±2,525)	4,100 (2,990–5,689)	–	4,473 (±2,785)	3,785 (2,692–5,592)	–	0.528
Neutrophils (%)	72 (±12.7)	73 (65–80)	–	69 (±13.5)	68 (61–79)	–	0.076
Lymphocytes (mmc)	1,013 (±517)	920 (640–1,300)	–	1,168 (±775)	925 (687–1,350)	–	0.111
Lymphocytes (%)	18 (±9.9)	16 (12–24)	—	20 (±11.4)	18 (11–28)	–	0.162
Monocytes (mmc)	368 (±168)	320 (250–450)		354 (±151)	325 (240–470)	–	0.562
C-reactive protein (mg/L)	11,645 (±133,032)	63,540 (22,375–160,770)	–	77,257 (±101,404)	34,900 (12,375–113,970)	–	**0.020**
D-dimer (mg/dl)	1,444 (±1,199)	1,268 (627–3,147)	–	1,193 (±1,212)	788 (484–1,770)	–	0.188
Albumin (mg/dl)	35.9 (±5.8)	37 (32.0–40)	–	38.1 (±5.4)	39.0 (34–42)	–	**0.008**
LDH (U/L)	358.9 (±1,923)	310 (242.3–419.3)	–	300.8 (±126)	272.0 (211–379)	–	**0.012**
CHARLSON index	2.7 (±2.3)	2 (1–4)	–	2.3 (±2.0)	2 (1–4)	–	0.313
Body Mass Index (BMI)	23 (±3)	–	–	24 (±2)	–	–	0.13
CURB-65	1 (±1)	1 (0–2)	–	1 (±1)	1 (0–2)	–	0.395
EXP CURB 65	2 (±1)	2 (1–3)	–	2 (±1)	2 (1–3)	–	0.108
PSI	76 (±31)	73 (54–91)	–	71 (±28)	69 (49–86)	–	0.211
CALL	9 (±3)	9 (7–11)	–	9 (±3)	9 (7–11)	–	0.869
Lenght of hospitalization (days)	18 (±13)	14 (8–23)	–	23 (±14)	20 (11–31)	–	**0.012**
Intensive care unit (ICU) hospitalization	–	–	24 (19)	–	–	16 (18)	0.847
History of CV disease	–	–	0 (0)	–	–	0 (0)	1.000
Death	–	–	34 (30)	–	–	10 (11)	** < 0.001**
Bloodstream infection (BSI)	–	–	14 (13)	–	–	7 (8)	0.211
Lung superinfections	–	–	9 (8)	–	–	8 (9)	0.904
Fungal infections	–	–	2 (2)	–	–	0 (0)	0.158

The two groups were comparable for Charlson comorbidity index and severity of SARS-CoV-2 related pneumonia. CRP concentrations were significantly higher initially in the group treated with BAT and bacteriotherapy. In the same group, LDH was significantly lower (*p* = 0.012). In the subjects treated only with BAT, the albumin was lower than in the group treated with BAT and bacteriotherapy.

### Mortality was Lower in Patients Treated With Bat Plus Oral Bacteriotherapy

**Figure 1:**
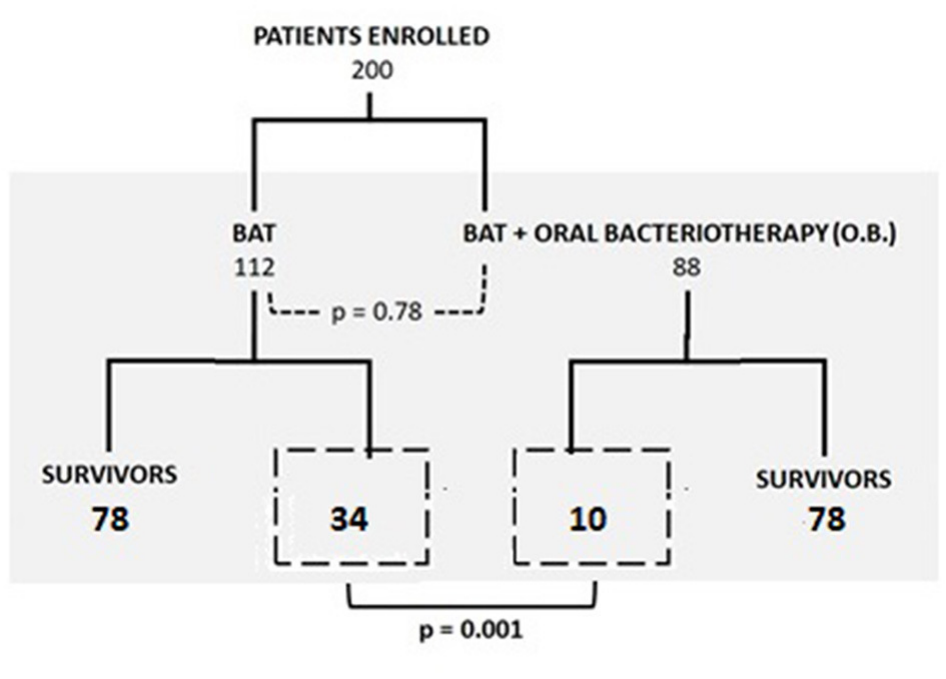
Mortality in wards and ICU in the two groups (BAT treatment vs. BAT and oral bacteriotherapy).

The primary endpoint was in-patient hospital crude mortality evaluated in each treatment group. Cumulative crude mortality was 22% (44 patients). Ten patients (11%) died in the group of patients treated with BAT plus oral bacteriotherapy vs. 34 (30%) subjects in the group of patients managed only with BAT (*p* < 0.001) (Figure 1).

**Figure 2:**
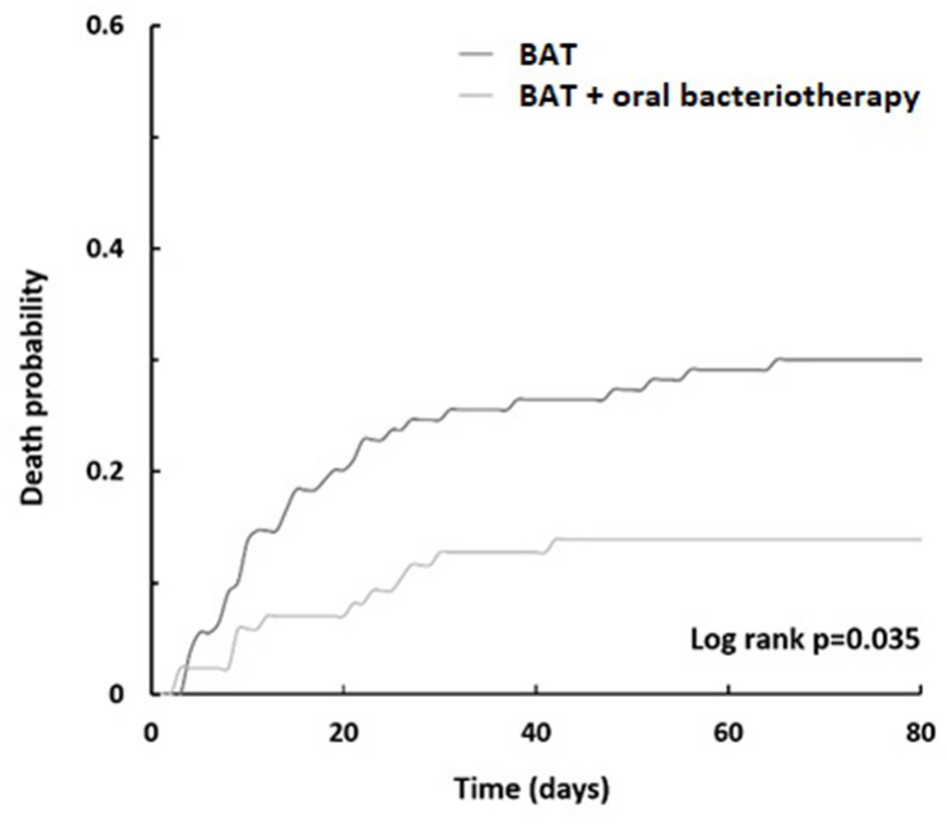
Death probability. Kaplan–Meier curves displaying BAT treatment vs. BAT and oral bacteriotherapy.

The significant reduction in risk of death present for patients treated with both BAT and oral bacteriotherapy was reconfirmed after adjustment for age, Charlson and CURB, CURB-65, PSI, and Call score with an OR of 0.28 (95% CI, 0.13–0.6, *p* = 0.001). The unweighted Kaplan–Meyer calculated showed the beneficial effect of combined BAT and oral bacteriotherapy on the parameter death probability (log rank *p* = 0.035 Figure 2). Moreover, the incidence of mortality related to the different BAT regimens used in the two groups was reported in Table 3.

**Table 3:** Summary of the combination of drugs used, degree of pneumonia severity, and incidence of mortality.

	**Therapy**	***n* (%)**	**CSI**	**CURB-65**	**EXP CURB-65**	**PSI**	**CALL**	**Incidence of mortality (%)**
BAT without oral bacteriotherapy (n = 112)	Single drug	**18 (16)**	**3 (0–8)**	**1 (0–2)**	**3 (0–4)**	**78 (15–138)**	**8.5 (4–16)**	**2 (2)**
	Hydroxycloroquine	13 (12)	2 (0**–**7)	1 (0**–**2)	3 (0**–**4)	64 (15**–**135)	7 (4**–**16)	2 (2)
	Lopinavir/r	0 (0)	–	**–**	**–**	**–**	**–**	0 (0)
	Tocilizumab	5 (5)	4 (0**–**8)	1 (1**–**2)	3 (2**–**4)	92 (49–138)	10 (5–12)	0 (0)
	Azithromycin	(0)	–	–	–	–	–	0 (0)
	Combination of 2 drugs	**49 (43)**	**2.5 (0**–**13)**	**1 (0**–**3)**	**2 (0**–**5)**	**75 (27**–**165)**	**8.5 (4**–**13)**	**17 (15)**
	Hydroxycloroquine, Tocilizumab	12 (11)	4.5 (0–8)	2 (0–3)	2.5 (1–5)	82.5 (35–149)	10 (7–12)	8 (7)
	Hydroxycloroquine, Lopinavir/r	12 (11)	1 (0–6)	1 (0–2)	2 (0–4)	54 (43–126)	7 (4–11)	3 (3)
	Hydroxycloroquine, Azithromycin	17 (15)	2 (0–13)	1 (0–3)	2 (0–5)	71 (27–165)	7 (4–13)	5 (4)
	Tocilizumab, Azithromycin	8 (7)	3 (0–7)	1 (0–2)	2.5 (0–4)	91 (29–151)	10 (4–13)	1 (1)
	Tocilizumab, Lopinavir/r	0 (0)	–	–	–	–	–	0 (0)
	Azithromycin, Lopinavir/r	0 (0)	–	–	–	–	–	0 (0)
	Combination of 3 drugs	**37 (33)**	**2 (0**–**6)**	**1 (0**–**2)**	**2 (0**–**5)**	**67 (19**–**120)**	**9 (4**–**11)**	**12 (11)**
	Hydroxycloroquine, Lopinavir/r, Tocilizumab	20 (18)	1.5 (0–4)	1 (0–2)	2 (0–5)	70.5 (39–118)	9 (4–11)	9 (8)
	Hydroxycloroquine, Lopinavir/r, Azithromycin	9 (8)	1 (0–6)	1 (0–2)	2 (1–3)	51 (39–87)	7 (5–11)	2 (2)
	Hydroxycloroquine, Tocilizumab, Azithromycin	8 (7)	2.5 (0–5)	1 (0–2)	2.5 (1–3)	79.5 (19–120)	10 (5–12)	2 (2)
	Combination of the 4 drugs	**8 (7)**	**4 (0**–**6)**	**2 (0**–**2)**	**3.5 (1**–**4)**	**90 (32**–**132)**	**10.5 (7**–**13)**	**3 (3)**
BAT plus oral bacteriotherapy (*n* = 88)	Bacteriotherapy and 1 drug	**13 (15)**	**4 (0**–**6)**	**1 (0–2)**	**1 (0–4)**	**83 (21–121)**	**9 (4–13)**	**2 (2)**
	Hydroxycloroquine	13 (15)	4 (0**–**6)	1 (0**–**2)	1 (0–4)	83 (21–121)	9 (4–13)	2 (2)
	Lopinavir/r	0 (0)	–	–	–	–	–	0 (0)
	Tocilizumab	0 (0)	–	–	–	–	–	0 (0)
	Azithromycin	0 (0)	–	–	–	–	–	0 (0)
	Bacteriotherapy and 2 drugs	**40 (45)**	**2 (0**–**5)**	**0 (0**–**3)**	**1.5 (0**–**5)**	**64 (18**–**111)**	**8 (4**–**12)**	**3 (3)**
	Hydroxycloroquine, Tocilizumab	13 (15)	2 (0–5)	1 (0–3)	2 (1–5)	67 (37–111)	8 (5–12)	2 (2)
	Hydroxycloroquine, Lopinavir/r	6 (7)	2 (0–5)	0 (0–2)	1.5 (1–4)	68 (39–108)	7.5 (7–12)	1 (1)
	Hydroxycloroquine, Azithromycin	20 (23)	2 (0–5)	0 (0–2)	1 (0–3)	64.5 (18–99)	7 (4–12)	0 (0)
	Tocilizumab, Azithromycin	1 (1)	2	2	2	56	9	0 (0)
	Bacteriotherapy and 3 drugs	**28 (32)**	**2 (0**–**6)**	**1 (0**–**3)**	**2 (0**–**5)**	**65 (32**–**111)**	**8 (5**–**13)**	**5 (6)**
	Hydroxycloroquine, Lopinavir/r, Tocilizumab	7 (8)	2 (0–5)	0 (0–2)	2 (1–4)	51 (32–83)	9 (7–12)	0 (0)
	Hydroxycloroquine, Lopinavir/r, Azithromycin	4 (4.5)	2 (0–6)	1 (0–2)	1 (0–3)	66 (23–111)	7 (5–10)	1 (1)
	Hydroxycloroquine, Tocilizumab, Azithromycin	17 (19)	3 (0–5)	1 (1–3)	3 (1–5)	79 (49–108)	9 (7–13)	4 (4.5)
	Bacteriotherapy and the 4 drugs	**3 (3)**	**2 (0**–**3)**	**2 (0**–**2)**	**1 (0**–**3)**	**54 (36**–**69)**	**10 (7**–**34)**	**0 (0)**

*The values in bold highlight statistically significant differences*.

**Figure 3:**
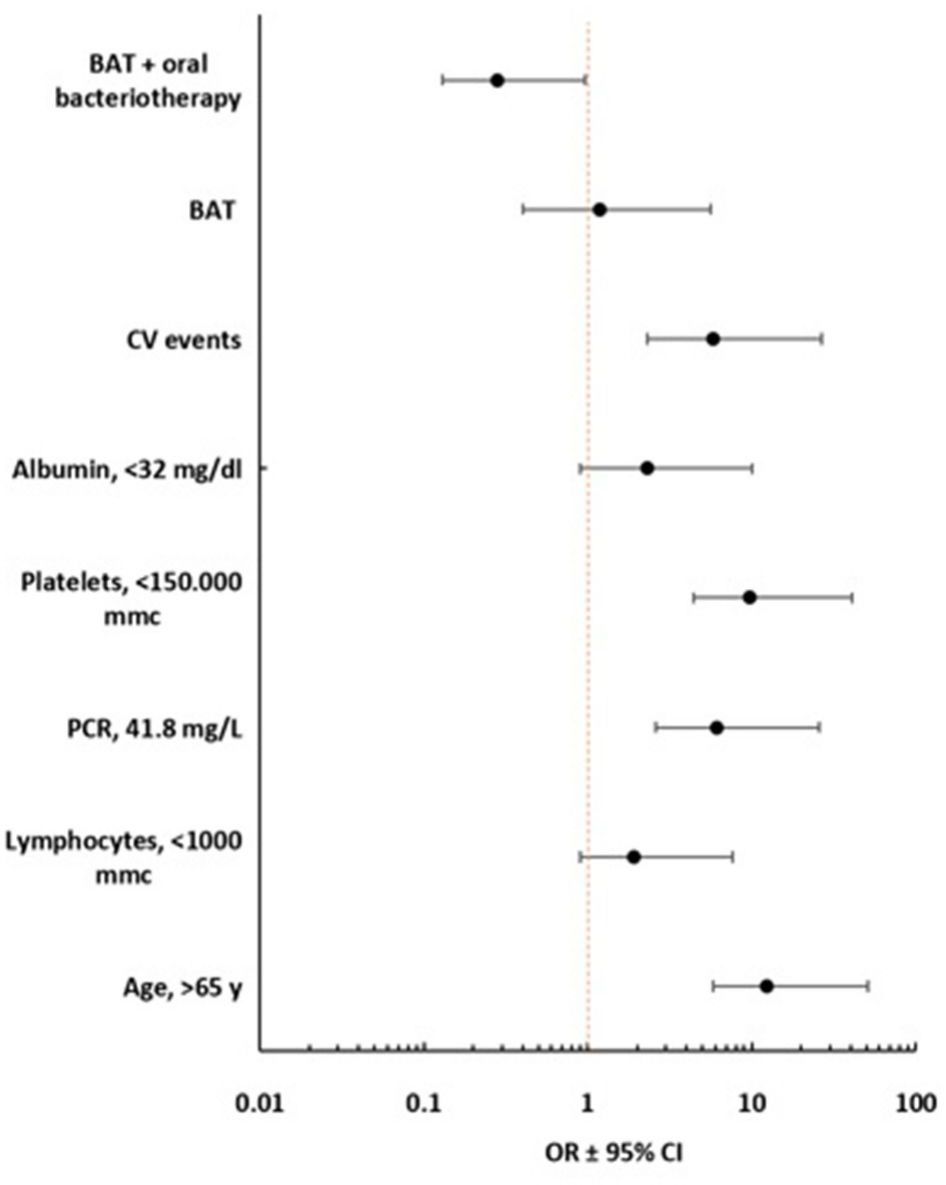
Forest plot multivariate analysis with 95% of Confidence interval (Lower, Upper).

By multivariate analysis, the age >65 years, CRP >41.8 mg/L, Platelets <150,000 mmc, and CV events were associated with the increased risk of mortality. Oral bacteriotherapy was an independent variable associated with a reduced risk for death (Figure 3).

### ICU Hospitalization

A severe worsening of cardio-respiratory conditions was the reason for the patient's transfer to ICU. These patients had a lower number of platelet and lymphocytes and a higher value of CRP than the patients staying in the ward (data not shown).

The need for ICU care was 21.4% (24/112) in BAT group and 18.1% (16/88) in BAT plus bacteriotherapy group. Patients who received BAT and oral bacteriotherapy had a similar risk of transfer in ICU when compared with the population who was receiving BAT alone (calculated by unadjusted Cox regression model (OR, 1.3, 95% CI 0.8–2.4, *p* = 0.270, vs. OR, 1.0. 95% CI 0.6–2.5, *p* = 0.554). These data were supported by various analyses aimed at controlling for confounding factors such as age, CRP, Charlson Comorbidity Index, exp CURB-65, CURB-65, PSI, Call, lymphocytes number, Platelet number, and the treatment effect (data not shown). No statistically significant differences were observed between the two groups regarding the incidence of bacterial and fungal superinfections in ICU. The patients treated with BAT had a hospitalization of 14 (IQR, 8–23) days vs. 20 (IQR, 11–31) days reported for patients receiving BAT and oral bacteriotherapy (*p* < 0.001).

### Safety

All patients were monitored for QT interval prolongation during treatment with hydroxychloroquine and azithromycin, as well as for the biochemical profile. Some patients reported mild gastric disturbances, not individually attributable to any of the drugs administered. No episodes of bacteremia attributable to the bacterial strains present in product utilized for bacteriotherapy were recorded.

## Discussion

Elderly subjects, immunocompromised patients, and people with comorbidities such as type 2 diabetes, heart disease, and vascular disorders are failing in the fight against COVID-19 (15). What is interesting to note is that dysbiosis is generally associated with these patients and the elderly. Several bacterial metabolites and bacterial fragments can vary the immune response in the lungs (the so-called “gut-lung” axis). The current hypothesis is that beneficial microorganisms in the digestive tract can play a significant role in determining the course of COVID-19 disease (16–18).

Given that the intestinal microbiota is adaptable and adjustable with diet, personalized dietary solutions can be deployed as a supplement to present-day routine therapies against COVID-19. We can achieve this goal by administering selected strains of lactobacilli and bifidobacteria to boost immunity and exert antiviral activities. The bacterial strains, present in the product delivered in this study, increase the production of both the nuclear factor erythroid 2p45 related to factor 2 (Nrf2) and its target Heme oxygenase-1 (HO-1) (6–19). Such molecules exert antiviral activity through a limitation of oxidative stress. Nrf2 and HO-1 have significant antiviral activity against a wide variety of viruses, including human immunodeficiency virus (HIV), influenza virus, respiratory syncytial virus, dengue virus, and Ebola virus, among others. Beneficial properties of HO-1 expression have been reported for viruses that cause lung disease (20–23).

Worldwide, it is estimated that the mortality rate for SARS-CoV-2 is around 6–7%. Different descriptive studies indicate an increased mortality rate in hospitalized patients, from 10 to 30%. For this study, mortality was 22%, reflecting a high prevalence of co-morbid diseases in patients with COVID-19 admitted to our Institution. Our study's death predictors included age ≥ 65 years old, CRP (>41.8 mg/L), platelets (<150,000 mmc), lymphocytes (<1,000 mmc), and cardiovascular problems. The cumulative probabilities calculated with Kaplan–Meyer's analyses for the entire population studied were 0.23 for deaths, 26 (16%) for transfer to intensive care, and 18 (22%) for hospitalization.

In a previous paper, we have reported a dramatic improvement in extrapulmonary symptomatology after 3 days of bacteriotherapy and a minor risk for mechanical ventilation (6). In this report, we confirm that few days after bacteriotherapy, the survival probability is significantly ameliorated. Moreover, the combined treatment BAT and bacteriotherapy is overall associated with a significant reduction in the mortality compared to the BAT group (10 = 11% vs. 34 = 30%; *p* < 0.001). We are not able to define if the observed positive effect of a specific oral bacteriotherapy can be explained by a direct effect on gut microbiota or an anti-viral activity or both. Anyway, as previously reported, oral bacteriotherapy administration could modulate the gut-lung axis, and possibly ameliorate the outcome of lung infection, by its biochemical and immunological profile that might trigger several protective biological functions (6).

According to our results, more drugs in combination does not mean a better therapeutic outcome for COVID-19 patients. The combinations Hydroxychloroquine with Lopinavir/r and/or Tocilizumab and/or Azithromycin were always less effective in terms of death rate to hydroxychloroquine alone. The key factor for the survival of the patient was oral bacteriotherapy, according to our observations. The “zero-mortality” target was achieved in 20 patients (23%) with the combination bacteriotherapy, hydroxychloroquine, and azithromycin and 1 patient (1%) who assumed bacteriotherapy, azithromycin, and tocilizumab.

This study's limitations are primarily related to the fact that it is the results of the observations from a single-center, and it is not prospective and not blinded. The only plausible explanation for the more extended hospitalization of patients treated with bacteriotherapy is a better survival (18 days BAT vs. 23 days BAT plus bacteriotherapy; *p* = 0.012). Another significant limitation is that the patients received a specific bacterial formulation with peculiar anti-inflammatory and antiviral activities. Therefore, the results are not directly transferable to different bacterial preparations (24–26). The two groups of patients enrolled were substantially comparable for clinical characteristics, but not for CRP, LDH, and albumin levels; anyway, the increased levels of the CRP and LDH (observed in the group treated with BAT and bacteriotherapy) suggest a more severe disease—connected with lung injury—and a worse prognosis but are not considered a main prognostic factor (27). Finally, the albumin levels were lower in the subjects treated only with BAT than in the other group, anyway, according to previous studies, only when albumin is below a specific range—i.e., <35g/L—the risks of venous and arterial thrombosis increase (28–32).

## Conclusion

Mounting evidence suggests SARS-CoV-2 may impact on host microbial flora and gut inflammation in patients affected by COVID-19. In particular, alterations of the gut microbiota composition might be a facilitating factor for an impaired immune response against the SARS-CoV-2 The possible effect of modulation of microbial flora and its implications are progressively assuming importance in COVID-19 research. Despite study's limitations previously highlighted and the need of large prospective trials to confirm the results reported, our studies suggest a possible role for oral bacteriotherapy in the management of patients hospitalized for COVID-19 pneumonia: in particular, the reduction in progression to severe disease and a lower mortality were highlighted in subjects in whom BAT is associated with oral bacteriotherapy.

## Consent for Publication

The participants provided their written consent for publication.

## Data Availability Statement

The original contributions presented in the study are included in the article/supplementary materials, further inquiries can be directed to the corresponding author/s.

## Ethics Statement

The studies involving human participants were reviewed and approved by Ethics Committee of Policlinico Umberto I—Sapienza University. The patients/participants provided their written informed consent to participate in this study.

## Author Contributions

GC and Gd'E contributed substantially to the conception and design of the study and interpretation and wrote the manuscript. CP, LS, GI, EC, LC, and FA contributed substantially to the acquisition of data and the analysis. CB and MM performed statistical analysis. FA, FR, and MV contributed substantially to data interpretation. MV, FP, CM, and Gd'E drafted or provided critical revision of the article and provided final approval of the version to publish. All authors contributed to the article and approved the submitted version.

## Conflict of Interest

The authors declare that the research was conducted in the absence of any commercial or financial relationships that could be construed as a potential conflict of interest.

## References

[1] Ramachandran P Onukogu I Ghanta S Gajendran M Perisetti A Goyal H. Gastrointestinal symptoms and outcomes in hospitalized COVID-19 patients. Dig Dis. (2020) 38:373–9. 10.1159/000509774 32599601 PMC7445385

[2] Nobel YR Phipps M Zucker J Lebwohl B Wang TC Sobieszczyk ME. Gastrointestinal symptoms and COVID-19: a case-control study from the United States. Gastroenterology. (2020). 159:373–5.e2. 10.1053/j.gastro.2020.04.017 32294477 PMC7152871

[3] Zhang J Yu M Tong S Liu LY Tang LV. Predictive factors for disease progression in hospitalized patients with coronavirus disease 2019 in Wuhan, China. J Clin Virol. (2020) 127:104392. 10.1016/j.jcv.2020.104392 32361327 PMC7187844

[4] Pouya F Imani Saber Z Kerachian MA. Molecular aspects of co-morbidities in COVID-19 infection. Arch Bone Jt Surg. (2020) 8(Suppl 1):226–30. 10.22038/abjs.2020.47828.2361 32607393 PMC7296607

[5] Viana SD Nunes S Reis F. ACE2 imbalance as a key player for the poor outcomes in COVID-19 patients with age-related comorbidities - Role of gut microbiota dysbiosis. Ageing Res Rev. (2020) 62:101123. 10.1016/j.arr.2020.101123 32683039 PMC7365123

[6] Chen P Lei J Wu Y Liu G Zhou B Liver impairment associated with disease progression in COVID-19 patients. Liver Int. (2020) 40:2308 10.1111/liv.14481 32294285 PMC7262073

[7] d'Ettorre G Ceccarelli G Marazzato M Campagna G Pinacchio C Alessandri F. Challenges in the management of SARS-CoV2 infection: the role of oral bacteriotherapy as complementary therapeutic strategy to avoid the progression of COVID-19. Front. Med. (2020) 7:389. 10.3389/fmed.2020.00389 32733907 PMC7358304

[8] World Health Organization (WHO) Laboratory Testing for 2019 Novel Coronavirus (2019-nCoV) in Suspected Human Cases. Interim guidance (2020). Available online at: https://www.who.int/publications/i/item/10665-331501 (accessed July 22, 2020).

[9] Società Italiana di Malattie Infettive e Tropicali (SIMIT) - Sezione Regione Lombardia 13 March (2020). Handbook for the Care of People With COVID-19 Disease, Version 2.0. (2020). Available online at: https://www.simit.org/news/11-vademecum-per-la-cura-delle-persone-con-malattia-da-covid-19 (accessed July 22, 2020).

[10] Charlson ME Pompei P Ales KL MacKenzie CR. A new method of classifying prognostic comorbidity in longitudinal studies: development and validation. J Chronic Dis. (1987) 40:373–83. 10.1016/0021-9681(87)90171-8 3558716

[11] Lim WS van der Eerden MM Laing R Boersma WG Karalus N Town GI. Defining community acquired pneumonia severity on presentation to hospital: an international derivation and validation study. Thorax. (2003) 58:377–82. 10.1136/thorax.58.5.377 12728155 PMC1746657

[12] Howell MW Donnino D Talmor Clardy P Ngo L Shapiro NI. Performance of severity of illness scoring systems in emergency department patients with infection. Acad Emerg Med. (2007) 14:709–14. 10.1197/j.aem.2007.02.036 17576773

[13] Liu JL Xu F Zhou H Wu X-j Shi L-x Lu R-q. Expanded CURB-65: a new score system predicts severity of community-acquired pneumonia with superior efficiency [published correction appears in *Sci Rep*. (2018) 8:47005]. Sci Rep. (2016) 6:22911. 10.1038/srep22911 26987602 PMC4796818

[14] Fine MJ Auble TE Yealy DM Hanusa BH Weissfeld LA Singer DE. A prediction rule to identify low-risk patients with community-acquired pneumonia. N Engl J Med. (1997) 336:243–50. 10.1056/NEJM199701233360402 8995086

[15] Ji D Zhang D Xu J Chen Z Yang T Zhao P. Prediction for progression risk in patients with COVID-19 pneumonia: the CALL score [published online ahead of print, 2020]. Clin Infect Dis. (2020) 71:1393–9. 10.1093/cid/ciaa414 32271369 PMC7184473

[16] Gao S Jiang F Jin W Shi Y Yang L Xia Y. Risk factors influencing the prognosis of elderly patients infected with COVID-19: a clinical retrospective study in Wuhan, China. Aging (Albany NY). (2020) 12:12504–16. 10.18632/aging.103631 32651993 PMC7377843

[17] Conte L Toraldo DM. Targeting the gut-lung microbiota axis by means of a high-fibre diet and probiotics may have anti-inflammatory effects in COVID-19 infection. Ther Adv Respir Dis. (2020) 14:1753466620937170. 10.1177/1753466620937170 32600125 PMC7328354

[18] Tulic MK Piche T Verhasselt V. Lung-gut cross-talk: evidence, mechanisms and implications for the mucosal inflammatory diseases. Clin Exp Allergy. (2016) 46:519–28. 10.1111/cea.12723 26892389

[19] Kalantar-Zadeh K Ward SA Kalantar-Zadeh K El-Omar EM Considering the effects of microbiome and diet on SARS-CoV-2 infection: Nanotechnology roles. ACS Nano. (2020) 14:5179–82. 10.1021/acsnano.0c03402 32356654

[20] Castelli V Angelo M Lombardi F Alfonsetti M Antonosante A Catanesi M. Effects of the probiotic formulation SLAB51 in *in vitro* and *in vivo* Parkinson's disease models. Aging (Albany NY). (2020) 12:4641–59. 10.18632/aging.102927 32155131 PMC7093198

[21] Hashiba T Suzuki M Nagashima Y Suzuki S Inoue S Tsuburai T. Adenovirus-mediated transfer of heme oxygenase-1 cDNA attenuates severe lung injury induced by the influenza virus in mice. Gene Ther. (2001) 8:1499–507 10.1038/sj.gt.3301540 11593363

[22] Espinoza JA León MA Céspedes PF Gómez RS Canedo-Marroquín G Riquelme SA. Heme oxygenase-1 modulates human respiratory syncytial virus replication and lung pathogenesis during infection. J Immunol. (2017) 199:212–23. 10.4049/jimmunol.1601414 28566367

[23] Tseng CK Lin CK Wu YH Chen YH Chen WC Young KC. Human heme oxygenase 1 is a potential host cell factor against dengue virus replication. Sci Rep. (2016) 6:32176. 10.1038/srep32176 27553177 PMC4995454

[24] Hill-Batorski L Halfmann P Neumann G Kawaoka Y. The cytoprotective enzyme heme oxygenase-1 suppresses Ebola virus replication. J Virol. (2013) 87:13795–802. 10.1128/JVI.02422-13 24109237 PMC3838215

[25] Ceccarelli G Scagnolari C Pugliese F Mastroianni CM d'Ettorre G. Probiotics and COVID-19. Lancet Gastroenterol Hepatol. (2020) 5:721–2. 10.1016/S2468-1253(20)30196-5 32673604 PMC7357989

[26] Infusino F Marazzato M Mancone M Fedele F Mastroianni CM Severino P. Diet supplementation, probiotics, and nutraceuticals in SARS-CoV-2 infection: a scoping review. Nutrients. (2020) 12:1718. 10.3390/nu12061718 32521760 PMC7352781

[27] Ceccarelli G Statzu M Santinelli L Pinacchio C Bitossi C Cavallari EN. Challenges in the management of HIV infection: update on the role of probiotic supplementation as a possible complementary therapeutic strategy for cART treated people living with HIV/AIDS. Expert Opin Biol Ther. (2019) 19:949–65. 10.1080/14712598.2019.1638907 31260331

[28] Han Y Zhang H Mu S Wei W Jin C Tong C. Lactate dehydrogenase, an independent risk factor of severe COVID- 19 patients: a retrospective and observational study. Aging. (2020) 12:11245–58. 10.18632/aging.103372 32633729 PMC7343511

[29] Toumazi D. Constantinou C. A fragile balance: the important role of the intestinal microbiota in the prevention and management of colorectal cancer. Oncology. (2020) 98:593–602. 10.1159/000507959 32604093

[30] Li N Ma WT Pang M Fan QL Hua JL. The commensal microbiota and viral infection: a comprehensive review. Front Immunol. (2019) 10:1551. 10.3389/fimmu.2019.01551 31333675 PMC6620863

[31] Violi F Cangemi R Romiti GF Ceccarelli G Oliva A Alessandri F. Is albumin predictor of mortality in COVID-19? Antioxid Redox Signal. (2020). 10.1089/ars.2020.8142. [Epub ahead of print]. 32524832

[32] Violi F Ceccarelli G Cangemi R Alessandri F D'Ettorre G Oliva A. Hypoalbuminemia, coagulopathy, and vascular disease in COVID-19. Circ Res.. (2020) 127:400–1. 10.1161/CIRCRESAHA.120.317173 32508261

